# Associations of sex on economic burden in patients with symptomatic obstructive hypertrophic cardiomyopathy: results from medical and pharmacy claims data

**DOI:** 10.3389/fcvm.2025.1463439

**Published:** 2025-04-07

**Authors:** Michael Butzner, Sanika Amonkar, Meiling Chen, Eros Papademetriou, Ravi Potluri, Xing Liu, Theodore Abraham

**Affiliations:** ^1^ Health Economics and Outcomes Research, Cytokinetics Incorporated, South San Francisco, CA, United States; ^2^ School of Medicine, University of California, San Francisco, CA, United States; ^3^ Health Economics and Outcomes Research, Putnam Associates, LLC, Boston, MA, United States

**Keywords:** economic burden, hypertrophic cardiomyopathy, obstructive, sex differences, real-world evidence

## Abstract

**Background:**

Previous studies of patients with symptomatic obstructive hypertrophic cardiomyopathy (oHCM) have reported worse clinical burden for female patients; whether this translates to an increase in healthcare resource use (HRU) and cost is unknown. Therefore, we evaluated the impact of sex on economic burden in symptomatic oHCM.

**Methods:**

Medical and pharmacy claims data were assessed from 2016 to 2021 to identify (ICD-10 code) adult patients with symptomatic oHCM in the United States. Generalized linear models were used to estimate HCM-related cost and generalized estimating equations for HRU [both reported as mean per-person-per-year (PPPY)] for healthcare categories: inpatient, outpatient, emergency room (ER), urgent care, and pharmacy. Cox proportional hazard regressions were used to compare differences in male and female patients with symptomatic HCM.

**Results:**

Among 9,490 patients with symptomatic oHCM, 5,309 (55.9%) were female. Female patients were older (64 ± 13 vs. 59 ± 14), with a higher Charlson Comorbidity Index (1.9 vs. 1.7) compared to males, respectively. After adjusting for patient characteristics, female patients had significantly greater number of HCM-related hospitalizations (0.24 vs. 0.20 PPPY, *p* = 0.0014), LOS (5.08 vs. 4.30 PPPY; *p* = 0.0235), number of outpatient visits (4.98 vs. 4.59 PPPY; *p* = 0.0387), and number of distinct drugs (0.59 vs. 0.55 PPPY; *p* = 0.0010), compared with males, respectively. In adjusted models, only HCM-related pharmacy costs were significant, with female patients having slightly higher costs compared to males ($70 vs. $61 PPPY; *p* = 0.0465). There were no significant differences in all-cause costs of care between male and female patients with oHCM.

**Conclusions:**

Female patients with symptomatic oHCM experience greater rates of HCM-related and all-cause hospitalizations and number of prescriptions, and HCM-related length of stay, outpatient visits, and pharmacy costs compared to male patients.

## Introduction

Hypertrophic Cardiomyopathy (HCM) is a chronic, progressive myocardial disorder defined by LV hypertrophy (1). An ECG-based epidemiologic study showed a disease prevalence of 1 case per 500 people in the general population, but a higher prevalence (1 case per 200) can be calculated when both clinical and genetic diagnoses are considered (2, 3). Using population-based methods, the estimated average prevalence of HCM around the world varies from 1 in 1,250 people in the US to a range of 1 in 1,372 people to 1 in 3,195 people in European countries (4–7). Approximately two-thirds of diagnosed HCM cases are obstructive HCM (oHCM) (8), and an estimated 50% of patients with oHCM are symptomatic (9). Previous studies have outlined the economic burden for patients with oHCM (9–11), but no evidence exists on the impact of sex on economic burden in this disease. Because female sex is associated with a higher risk of HCM-related events, HCM-related death, major cardiovascular events, cardiovascular death, noncardiovascular death, and all-cause mortality (12), it is important to understand whether this translates to an increase in healthcare resource use (HRU) and cost. The objective of this study was to evaluate the impact of sex on HRU and costs in patients with symptomatic oHCM using a large, national database of medical and pharmacy administrative claims.

## Methods

### Data source and study design

The study is a retrospective analysis of longitudinal medical and pharmacy claims data from Symphony Integrated Dataverse (IDV) database. The IDV is an open claims administrative health claims database that contains prescription, medical, and hospital claims across the US for all payment types, including commercial plans, Medicare Part D, cash, assistance programs, and Medicaid. The IDV contains over 10 billion deidentified prescriptions claims linked to over 280 million unique patients with an average of 5 years of prescription drug history. These prescription drug claims are linked to hospital and physician practices claims with medical procedure (i.e., current procedural terminology [CPT] and diagnosis codes [International Classification of Disease Tenth Revision (ICD-10)] for nearly 180 million patients. The full database includes claims from over 65,000 pharmacies, 1,500 hospitals, 800 outpatient facilities, and 80,000 physician practices across the US, capturing approximately 75% of the total prescriptions dispensed in the US. The IDV database was selected due to its longitudinal nature and comprehensive coverage of claims required to meet the study objectives. The distribution of Symphony Health patients across census regions is very similar to that of the US population.

Patients of interest were identified from January 1st, 2017 to April 30th, 2021, with a 12-month index period. The first relevant ICD-10 claim of HCM diagnosis was considered as the index diagnosis date. The date of first treatment with a beta-blocker, calcium channel blocker, disopyramide, or a procedure of interest (e.g., alcohol septal ablation, septal myectomy, pacemaker) after diagnosis date was considered as the index treatment date. Patient demographics and clinical characteristics were captured at baseline and costs and HRU were captured over the follow-up period. The data used in this study were de-identified in compliance with the Health Insurance Portability and Accountability Act.

### Patient selection

**Figure 1:**
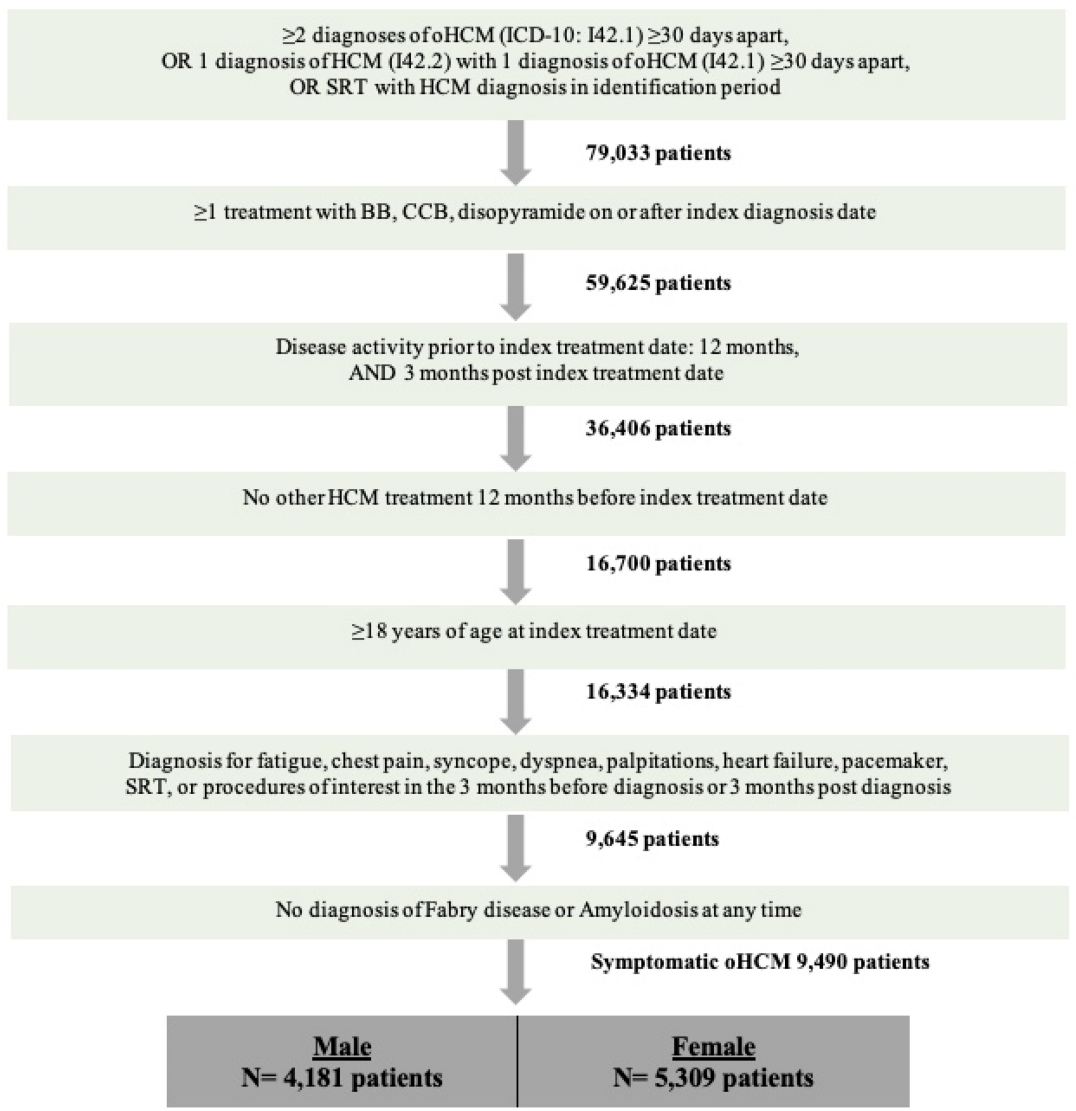
Patient selection criteria.

The patient population for this study consisted of prevalent patients with oHCM identified from the IDV (Figure 1). Adult (Age ≥ 18 years at index treatment date) patients with oHCM who met the following criteria were included in this study (1) ≥two claims of oHCM (ICD-10 diagnosis code: I42.1) at least 30 days apart, or (2) one diagnosis of HCM (ICD-10 diagnosis code: I42.2) along with either a diagnosis of oHCM (ICD-10 diagnosis code: I42.1) at least 30 days apart or a septal reduction therapy procedure any time after HCM diagnosis. Patients were required to have 12 months of activity prior to index treatment date, 3 months of activity post index treatment date, and be symptomatic. Patients were categorized as symptomatic if they had a diagnosis for fatigue, chest pain, syncope, dyspnea, heart failure, palpitations, insertion of a pacemaker, or septal reduction therapy, in the 3 months before or after their index diagnosis date. We excluded patients with a diagnosis of Fabry disease, amyloidosis, and patients with HCM treatment in the 12 months prior to index treatment date (i.e., patients were treatment-naïve at index treatment date). Patients were followed from their index treatment date until the end of index treatment due to either (1) discontinuation, (2) treatment switch, (3) treatment augmentation, or (4) end of activity in the database.

### Patient characteristics and study outcomes

Patient demographics and comorbidities, included the Charlson Comorbidity Index, were calculated 12 months prior to index date (excluding index date). All-cause and HCM-related HRU and costs were evaluated. Healthcare resource utilization and costs were defined for inpatient admissions, outpatient visits, ER visits, urgent care visits, other visits, and pharmacy costs (HCM-related prescriptions and non-HCM related prescriptions). HCM-related outcome variables included in this analysis were predefined based on the AHA/ACC treatment guidelines and confirmed by clinical experts who care for patients with HCM (13). Regarding the financial data available in the IDV, final adjudicated costs were only available for all-cause related prescriptions. For all other cost categories including HCM-related prescriptions, the amount billed by the payer (charged amount) is reported.

### Statistical analysis

Descriptive statistics were presented as means and standard deviation (SD), median and interquartile range (IQR) for continuous variables, and frequencies and proportions for categorical, and dichotomous variables. Baseline characteristics were compared with Chi-squared test for categorical variables or Kruskal–Wallis test for continuous variables. Systematic differences between sex were evaluated by Kruskal–Wallis for continuous variables and Chi-square tests for categorical variables. Generalized linear models with a gamma distribution and log link clustered on the patient was used to estimate per-person-per-year (PPPY) costs and generalized estimating equations with a negative binomial distribution clustered on the patient was used to estimation PPPY visits. The mean difference in PPPY costs/visits, 95% confidence intervals (CIs), and *p*-values were estimated for the patient cohort and by sex. These estimates were evaluated for each healthcare setting (inpatient, outpatient, ER, urgent care, other medical visits, and prescriptions).

An adjusted analysis of all-cause and HCM related HRU and costs was conducted. This included generalized linear models to estimate adjusted healthcare costs and generalized estimating equations to estimate adjusted healthcare visits. Covariates in the adjusted models were selected based on differences seen in a univariate analysis of baseline variables between males and females (Table 1). These included the baseline patient values of age, region, insurance type, hypertension, atrial fibrillation and flutter, heart failure, ventricular fibrillation, ventricular tachycardia, chronic pulmonary disease, obesity, valvular disease, stress cardiomyopathy, and coronary artery disease. Missing or unavailable data were not included in the analyses.

## Results

### Patient population

Among 9,490 patients with symptomatic oHCM, 5,309 (55.9%) were female and male patients had longer length of follow-up time (Table 1). Female patients were older (64 ± 13 vs. 59 ± 14; *p* < 0.0001) compared to males, respectively. Regardless of sex, the majority of patients in this cohort were 65 years of age or older (*N* = 4,726, 99.6%). Female patients also had a higher Charlson Comorbidity Index (1.9 vs. 1.7; *p* < 0.0001) compared to males, respectively. Baseline patient clinical characteristics for this cohort of patients with symptomatic oHCM are summarized in Table 2.

**Table 1:** Baseline patient demographics.

Characteristic	All patients (*N* = 9,490)		Male (*N* = 4,181; 44.1%)		Female (*N* = 5,309; 55.9%)		*p*-value
Age, years *n* (%)							
Mean (SD)	61.8	14.1	58.8	14.3	64.3	13.4	<0.0001
Median (IQR)	64.0	20.0	61.0	20.0	67.0	19.0	<0.0001
18–34	545	5.7%	334	8.0%	211	4.0%	<0.0001
35–44	645	6.8%	334	8.0%	311	5.9%	<0.0001
45–54	1,304	13.7%	740	17.7%	564	10.6%	<0.0001
55–64	2,270	23.9%	1,125	26.9%	1,145	21.6%	<0.0001
65+	4,726	99.6%	1,648	39.4%	3,078	58.0%	<0.0001
Year of index treatment, *n* (%)							
2017	1,885	19.9%	792	18.9%	1,093	20.6%	0.0462
2018	2,256	23.8%	1,025	24.5%	1,231	23.2%	0.1312
2019	2,319	24.4%	1,025	24.5%	1,294	24.4%	0.8731
2020	1,917	20.2%	864	20.7%	1,053	19.8%	0.3170
2021	1,113	11.7%	475	11.4%	638	12.0%	0.3238
Region, *n* (%)							
Northeast Region	2,298	24.2%	1,104	26.4%	1,194	22.5%	<0.0001
North Central Region	2,474	26.1%	1,087	26.0%	1,387	26.1%	0.8889
South Region	3,411	35.9%	1,428	34.2%	1,983	37.4%	0.0013
West Region	1,273	13.4%	546	13.1%	727	13.7%	0.3678
Unknown Region	34	0.4%	16	0.4%	18	0.3%	0.7239
Insurance type, *n* (%)							
Cash	439	4.6%	214	5.1%	225	4.2%	0.0427
Commercial	1,806	19.0%	940	22.5%	866	16.3%	<0.0001
Employer Group	701	7.4%	334	8.0%	367	6.9%	0.0467
Medicaid	1,138	12.0%	516	12.3%	622	11.7%	0.3517
Medicare	3,668	38.7%	1,273	30.4%	2,395	45.1%	<0.0001
PBM	679	7.2%	360	8.6%	319	6.0%	<0.0001
Government	34	0.4%	20	0.5%	14	0.3%	0.0823
Processors	25	0.3%	11	0.3%	14	0.3%	0.9954
Third party administrator	26	0.3%	18	0.4%	8	0.2%	0.0096
Unspecified	973	10.3%	495	11.8%	478	9.0%	<0.0001
Workers compensation	1	0.0%	0	0.0%	1	0.0%	–
Length of follow-up period							
Mean (SD)	425.2	450.5	451.9	461.8	404.2	440.3	<0.0001
Median (SD)	227.0	576.0	248.0	636.0	210.0	530.0	<0.0001

IQR, interquartile range; SD, standard deviation.

**Table 2:** Baseline patient clinical characteristics.

Characteristic	All patients (*N* = 9,490)		Male (*N* = 4,181; 44.1%)		Female (*N* = 5,309; 55.9%)		*p*-value
CCI score							
Mean (SD)	1.8	1.9	1.7	1.9	1.9	1.9	<0.0001
Median (IQR)	2.0	3.0	2.0	3.0	2.0	3.0	<0.0001
CCI Score category, *n* (%)							
0	3,522	37.1%	1,685	40.3%	1,837	34.6%	<0.0001
1	1,029	10.8%	388	9.3%	641	12.1%	<0.0001
2	2,158	22.7%	1,005	24.0%	1,153	21.7%	0.0074
3	1,218	12.8%	467	11.2%	751	14.1%	<0.0001
4	759	8.0%	292	7.0%	467	8.8%	0.0012
5+	804	8.5%	344	8.2%	460	8.7%	0.4480
Number of patients with any comorbidity, *n* (%)	8,798	92.7%	3,830	91.6%	4,968	93.6%	0.0002
Cardiac arrhythmia	5,144	54.2%	2,426	58.0%	2,718	51.2%	<0.0001
Atrial fibrillation	2,570	27.1%	1,226	29.3%	1,344	25.3%	<0.0001
Atrial flutter	505	5.3%	295	7.1%	210	4.0%	<0.0001
Ventricular fibrillation	139	1.5%	84	2.0%	55	1.0%	<0.0001
Ventricular tachycardia	1,168	12.3%	665	15.9%	503	9.5%	<0.0001
Supraventricular tachycardia	567	6.0%	232	5.5%	335	6.3%	0.1204
Chronic pulmonary disease	2,401	25.3%	825	19.7%	1,576	29.7%	<0.0001
Hypertension	6,535	68.9%	2,767	66.2%	3,768	71.0%	<0.0001
Hypertension (2 diagnosis)	3,939	41.5%	1,570	37.6%	2,369	44.6%	<0.0001
Congestive heart failure	4,037	42.5%	1,711	40.9%	2,326	43.8%	0.0047
Renal failure	1,360	14.3%	613	14.7%	747	14.1%	0.4145
Obesity	2,152	22.7%	877	21.0%	1,275	24.0%	0.0004
Diabetes	2,297	24.2%	984	23.5%	1,313	24.7%	0.1767
Diabetes complicated	1,390	14.6%	616	14.7%	774	14.6%	0.8328
Diabetes uncomplicated	1,937	20.4%	816	19.5%	1,121	21.1%	0.0551
Valvular disease	3,945	41.6%	1,570	37.6%	2,375	44.7%	<0.0001
Stress cardiomyopathy	46	0.5%	7	0.2%	39	0.7%	<0.0001
Stroke	468	4.9%	200	4.8%	268	5.0%	0.5547
Dyslipidemia	4,974	52.4%	2,174	52.0%	2,800	52.7%	0.4715
Coronary artery disease	3,177	33.5%	1,500	35.9%	1,677	31.6%	<0.0001

Patients with hypertension were required to have ≥2 diagnoses at least 30 days apart to confirm accuracy of diagnosis and rule out misdiagnosis of another cardiovascular disease.

IQR, interquartile range; SD, standard deviation.

### Healthcare resource utilization

After adjusting for patient characteristics, female patients had significantly greater number of HCM-related hospitalizations (0.24 vs. 0.20 PPPY, *p* = 0.0014), LOS (5.08 vs. 4.30 PPPY; *p* = 0.0235), number of outpatient visits (4.98 vs. 4.59 PPPY; *p* = 0.0387), and number of distinct drugs (0.59 vs. 0.55 PPPY; *p* = 0.0010), compared with males, respectively (Table 3). Additionally, female patients had significantly greater number of all-cause hospitalizations (0.96 vs. 0.82 PPPY, *p* = 0.0002) and number of distinct drugs (7.34 vs. 6.31 PPPY; *p* < 0.0001), after adjusting for patient characteristics (Table 4). Unadjusted HCM-related and all-cause HRU are presented in Supplementary Table S1 and Supplementary Table S2, respectively.

**Table 3:** Adjusted HCM-related healthcare resource utilization and costs.

Characteristic	All patients (*n* = 9,490)	Male (*n* = 4,181)	Female (*n* = 5,309)	*p*-value
Total cost, PPPY (95% CI)	$15,224 ($10,552–$21,966)	$15,071 ($10,332–$21,982)	$15,333 ($10,626–$22,126)	0.7629
Hospitalizations				
Patients with a hospitalization, *n* (%)	1,468 (15.5%)	637 (15.2%)	831 (15.7%)	
Number of hospitalizations, PPPY (95% CI)	0.23 (0.14–0.38)	0.20 (0.12–0.34)	0.24 (0.14–0.41)	0.0014
Hospitalization costs $ PPPY (95% CI)	$1,161 ($640–$2,107)	$1,121 ($611–$2,056)	$1,172 ($643–$2,136)	0.6617
Length of stay, per hospitalization Mean days (95% CI)	4.98 (3.67–6.74)	4.30 (3.2–5.78)	5.08 (3.78–6.83)	0.0235
Outpatient visits				
Patients with an outpatient visit, (%)	5,328 (56.1%)	2,475 (59.2%)	2,853 (53.7%)	
Number of outpatient visits PPPY (95% CI)	4.82 (3.6–6.44)	4.59 (3.42–6.17)	4.98 (3.72–6.66)	0.0387
Outpatient costs $ PPPY (95% CI)	$12,635 ($8,174–$19,531)	$12,758 ($8,138–$19,999)	$12,536 ($8,114–$19,368)	0.8053
Emergency room visits				
Patients with ER visit, *n* (%)	948 (10.0%)	421 (10.1%)	527 (9.9%)	
Number of ED visits	0.13 (0.06–0.29)	0.12 (0.05–0.27)	0.13 (0.06–0.3)	0.2470
ED costs	$146 ($56–$377)	$128 ($49–$331)	$150 ($58–$388)	0.1660
Urgent Care [n (PPPY), 95% CI]^†^				
Patients with UC visits, *n* (%)	4,635 (48.8%)	2,123 (50.8%)	2,512 (47.3%)	
Number of UC visits	2.98 (2.28–3.89)	2.86 (2.19–3.75)	3.05 (2.33–4)	0.0729
UC costs	$847 ($640–$1,122)	$825 ($622–$1,095)	$861 ($649–$1,142)	0.3040
Pharmacy [n (PPPY), 95% CI]^†^				
Patients with at least one pharmacy record, *n* (%)	8,372 (88.2%)	3,618 (86.5%)	4,754 (89.5%)	
Number of distinct drugs	0.57 (0.48–0.69)	0.55 (0.46–0.66)	0.59 (0.49–0.71)	0.0010
Pharmacy costs	$65 ($45–$95)	$61 ($41–$91)	$70 ($48–$102)	0.0465

Healthcare costs are presented as PPPY $USD 2022. Healthcare resource utilization is presented as PPPY.

CI, confidence interval; HCM, hypertrophic cardiomyopathy; ER, emergency room; PPPY, per person per year; UC, urgent care. Costs, visits, and length of stay were adjusted with the following covariates: Age, region, insurance type (commercial vs. non-commercial), atrial fibrillation, atrial flutter, ventricular fibrillation, ventricular tachycardia, chronic pulmonary, disease, hypertension, obesity, valvular disease, stress cardiomyopathy, coronary artery disease.

### Healthcare costs

Among symptomatic oHCM patients, total all-cause costs were greater compared to HCM-related costs, respectively ($51,835 vs. $23,048).     In adjusted models, only HCM-related pharmacy costs were significant, with female patients having slightly higher costs compared to males ($70 vs. $61 PPPY; *p* = 0.0465; Table 3). There were no significant differences in all-cause cost of care between male and female patients with oHCM (Table 4). Unadjusted HCM-related and all-cause costs are presented in Supplementary Table S1, and no significant differences in any all-cause costs categories between male and female patients with symptomatic oHCM (Supplementary Table S2).

**Table 4:** Adjusted All-cause healthcare resource utilization and costs.

Characteristic	All patients (*n* = 9,490)	Male (*n* = 4,181)	Female (*n* = 5,309)	*p*-value
Total cost, PPPY (95% CI)	$53,529 ($39,490–$72,559)	$53,762 ($39,357–$73,441)	$53,397 ($39,195–$72,744)	0.9140
Hospitalizations				
Patients with a hospitalization, *n* (%)	2,862 (30.2%)	1,228 (29.4%)	1,634 (30.8%)	
Number of hospitalizations, PPPY (95% CI)	0.90 (0.66–1.22)	0.82 (0.6–1.11)	0.96 (0.71–1.3)	0.0002
Hospitalization costs $ PPPY (95% CI)	$4,022 ($2,528–$6,398)	$3,950 ($2,467–$6,326)	$4,050 ($2,532–$6,479)	0.7427
Length of stay, per hospitalization Mean days (95% CI)	4.59 (3.81–5.54)	4.34 (3.59–5.23)	4.70 (3.88–5.69)	0.1373
Outpatient visits				
Patients with an outpatient visit, (%)	7,815 (82.3%)	3,461 (82.8%)	4,354 (82.0%)	
Number of outpatient visits PPPY (95% CI)	14.45 (11.45–18.24)	13.99 (11.04–17.72)	14.80 (11.71–18.71)	0.1040
Outpatient costs $ PPPY (95% CI)	$37,693 ($25,731–$55,215)	$38,215 ($25,788–$56,632)	$37,382 ($25,358–$55,108)	0.7889
Emergency room visits				
Patients with ER visit, *n* (%)	2,665 (28.1%)	1,148 (27.5%)	1,517 (28.6%)	
Number of ED visits	0.70 (0.46–1.07)	0.68 (0.45–1.05)	0.71 (0.47–1.09)	0.4820
ED costs	$1,175 ($710–$1,946)	$1,137 ($684–$1,890)	$1,202 ($724–$1,995)	0.4374
Urgent Care [n (PPPY), 95% CI]^†^				
Patients with UC visits, *n* (%)	6,697 (70.6%)	2,965 (70.9%)	3,732 (70.3%)	
Number of UC visits	7.03 (5.73–8.63)	6.84 (5.57–8.4)	7.17 (5.83–8.81)	0.0647
UC costs	$1,845 ($1,459–$2,333)	$1,845 ($1,459–$2,333)	$1,845 ($1,455–$2,340)	0.9895
Pharmacy [n (PPPY), 95% CI]^†^				
Patients with at least one pharmacy record, *n* (%)	9,026 (95.1%)	3,952 (94.5%)	5,074 (95.6%)	
Number of distinct drugs	6.94 (5.9–8.16)	6.31 (5.36–7.44)	7.34 (6.24–8.63)	<0.0001
Pharmacy costs	$2,474 ($1,682–$3,639)	$2,588 ($1,753–$3,820)	$2,388 ($1,619–$3,523)	0.2507

Healthcare costs are presented as PPPY $USD 2022. Healthcare resource utilization is presented as PPPY.

CI, confidence interval; HCM, hypertrophic cardiomyopathy; ER, emergency room; PPPY, per person per year; UC, urgent care. Costs, visits, and length of stay were adjusted with the following covariates: Age, region, insurance type (commercial vs. non-commercial), atrial fibrillation, atrial flutter, ventricular fibrillation, ventricular tachycardia, chronic pulmonary, disease, hypertension, obesity, valvular disease, stress cardiomyopathy, coronary artery disease.

## Discussion

To our knowledge, this is the first study to evaluate the impact of sex on HRU and costs in patients with symptomatic oHCM. Using a large, national database of medical and pharmacy administrative claims, we found that female patients with symptomatic oHCM, after adjusting for baseline patient characteristics, experience greater HCM-related and all-cause hospitalizations and prescriptions, and HCM-related length of stay and outpatient visits compared to male patients. Furthermore, female patients had greater HCM-related pharmacy costs, but there were no differences in all-cause costs of care by sex in symptomatic oHCM patient. Thus, suggesting that significant increases in costs of care for this cohort of patients may potentially be attributable to the specific nature of their symptomatic oHCM.

While previous investigations have analyzed the economic burden of patients with HCM (9, 10, 14), including invasive procedures like septal reduction therapy (11, 15–17), this is the first population-based study to evaluate the impact of sex on HRU and costs among patients with symptomatic oHCM. In a previous population-based study, Butzner et al. (2022) used a national medical and pharmacy claims database in the United States to evaluate HCM-related and all-cause economic burden for patients with oHCM (10). They found that costs related to oHCM increased from $5,968 to $20,290 at 1-year follow-up after oHCM diagnosis ($23,048 in the current analysis), driven mostly by inpatient hospitalizations and surgical costs (10). Additionally, in their cohort of oHCM patients, 27% had an inpatient hospitalization due to their HCM (10), compared to 15.5% of patients in the present study having an inpatient hospitalization. Our study extends upon these findings to show that when factoring in sex in adjusted models, female patients with symptomatic oHCM experience greater HRU compared to male counterparts.

Prior to this investigation, a single study used medical and pharmacy claims data to evaluate sex differences in oHCM treatment and cardiovascular outcomes among patients with oHCM (18). Regarding treatment, they found women were less likely to be prescribed HCM-related treatments including beta blockers and anticoagulants (18). In contrast, we found that female patients with symptomatic oHCM were more likely to have an HCM-related and all-cause prescription fill, with 89.5% of patients receiving a prescription to treat their oHCM. However, the present analysis evaluated HCM prescriptions as an inclusive category of all HCM prescriptions, without separating specific HCM medications to test differences. We extend upon these clinical differences among sex to report that after adjusting for baseline patient characteristics, female patients with symptomatic oHCM are prescribed more HCM-related and all-cause prescriptions to treat their disease compared with male patients.

The impact of comorbidities in oHCM (including by sex) is important to consider for the present analysis as female patients presented with greater comorbidity burden and increased HRU. For example, hemodynamic disruptions due to obstruction in HCM can be further exacerbated by systolic anterior motion of the mitral valve and are a critical component of disease development (19). Previous studies show that oHCM patients commonly have additional structural abnormalities of the sub-mitral valve (20, 21), with a significant difference in female and male patients (44.7% vs. 37.6, respectively) in the present analysis having valvular disease. Concomitant interventions to account for these additive comorbidities are associated with worse clinical outcomes including in- hospital death, adverse in-hospital events, and 30-day readmission (22). Further investigation is needed to understand whether differences in HRU and costs are driven primarily by oHCM or comorbidities, and if there are difference across sex.

It is also important to consider the present results in context of a contemporary population of HCM. In a recent study of a large, global registry of patients with HCM, Canepa et al. (2020) found that age of HCM diagnosis increased significantly over time with a stable male-to-female ratio, suggesting that evolving HCM populations include progressively greater representation of older patients (23). Socioeconomic factors, such as age, may impact patients' healthcare costs. While it is plausible to hypothesize that economic burden for patients increase with age, a retrospective analysis of 5,129 patients with oHCM found that being aged 18–39 years was associated with increased HCM-related healthcare costs (total, medical, office visit, outpatient visit, emergency room), compared to older categorical age groups (24). Additionally, the current findings must take into account a longer length of follow-up for male patients. Further investigation is needed to evaluate time-varying impact of both age and sex on costs of care and HRU in patients with HCM.

This study provides benchmark economic data on the impact of sex on the costs of care for patients with symptomatic oHCM. Female patients experienced greater HRU and pharmacy costs due to their HCM compared to male patients with symptomatic oHCM, after adjusting for patient characteristics. Healthcare costs and resource utilization have not been detailed in previous (13) and current (21) recommended guidelines for the management of patients with HCM, including the impact of sex on clinical and economic burden. The results from this analysis can be used to emphasize and bring awareness to sex differences in resource utilization and costs of care for symptomatic oHCM, highlighting the clinical importance of sex-based differences for diagnosis and management in HCM patients. Lastly, regarding economic differences in HCM prescription fills and increased utilization among female patients, the impact of emerging therapies, indicated for patients with symptomatic oHCM (25–28), on HRU and costs of care for patients with symptomatic oHCM should be evaluated.

### Limitations

This study is subject to several limitations, which are common across claims database analyses. The diagnoses, comorbidities, HRU and costs of patients with HCM were identified based on ICD-10-CM diagnosis code. The presence of a diagnosis code on a medical claim does not necessarily indicate a positive presence of disease because the medical record may have been incorrectly coded or included as a rule-out criterion rather than the actual disease. Also, diagnosis codes only signify the presence of the disease and do not detail the characteristics or the nature of the disease as you would find in electronic medical record data. This limitation was overcome by requiring eligible patients to have at least two claims with diagnosis codes for HCM. It was also ensured that generic codes, such as codes for “other cardiomyopathy” and “unspecified cardiomyopathy,” which could be used for HCM, were not included in the identification of HCM patients for the study.

Diagnosis codes were used to identify patients with symptomatic oHCM, and a combination of symptoms, comorbidities, and procedures was used to identify symptomatic oHCM in this study. Since the claims database does not have a record of all symptoms of a patient, and some of the symptoms could be attributed to comorbidities, there is the possibility of difference in actual proportion of patients with symptomatic oHCM and the estimates in this study. Additionally, due to restrictions of the database regarding HIPAA policies, we are unable to report separate costs for surgical procedures including septal reduction therapy. Furthermore, without patient level medical record data, the question remains whether these differences in HRU (including adjusted analyses) are driven primarily by HCM or the additive comorbidity burden. Lastly, the definition of costs in this analysis was charges, meaning the amount billed by the payer. This may not be reflective of what a patients pays for their cost of care, but what the charged amount is by the payer in the U.S. healthcare system.

## Conclusions

In this large, national cohort of symptomatic oHCM patients, adjusted models report that female patients with symptomatic oHCM experienced greater rates of HCM-related and all-cause hospitalizations and number of prescriptions, and HCM-related length of stay, outpatient visits, and pharmacy costs compared to male patients. These findings highlight the clinical importance of sex-based differences for diagnosis and management in HCM patients and warrant inclusion in current HCM treatment guidelines. Future research on the impact of emerging therapies on HRU and cost of care for patients with symptomatic oHCM should be evaluated.

## Data Availability

The original contributions presented in the study are included in the article/Supplementary Material, further inquiries can be directed to the corresponding author.
