# Supplementary material for: Associations of sex on economic burden in patients with symptomatic obstructive hypertrophic cardiomyopathy: results from medical and pharmacy claims data
Source: Front Cardiovasc Med. 2025 Apr 7;12:1463439. doi: 10.3389/fcvm.2025.1463439 (PMC12009858; doi:10.3389/fcvm.2025.1463439)
Supplement: Supplementary file 1 [file Datasheet1.pdf]

Supplementary Material

1. **Supplementary Table 1. Unadjusted HCM-Related Healthcare Resource Utilization and Costs**
2. **Supplementary Table 2. Unadjusted All-Cause Healthcare Resource Utilization and Costs**
3. **Supplementary Table 3. All-cause cost models result**
4. **Supplementary Table 4. All-cause resource models result**
5. **Supplementary Table 5. HCM related cost models result**
6. **Supplementary Table 6. HCM related resource use models result**

Supplementary Table 1. Unadjusted HCM-Related Healthcare Resource Utilization and Costs

	**Overall (n=9,490)**	**Male (n=4,181)**	**Female (n=5,309)**	***p*-value**
**Total cost, PPPY (95% CI)**	$19,913 ($18,895-$20,987)	$21,580 ($20,066-$23,207)	$18,525 ($17,174-$19,982)	0.0044
**Hospitalizations**				
Patients with a hospitalization, n (%)	1,468 (15.5%)	637 (15.2%)	831 (15.7%)	
Number of hospitalizations, PPPY (95% CI)	0.20 (0.19-0.22)	0.19 (0.17-0.2)	0.22 (0.2-0.24)	0.0059
Hospitalization costs $ PPPY (95% CI)	$2,695 ($2,489-$2,919)	$2,585 ($2,301-$2,904)	$2,791 ($2,503-$3,114)	0.3458
Length of stay, per hospitalization Mean days (95% CI)	5.12 (4.78-5.49)	4.66 (4.3-5.06)	5.46 (4.94-6.05)	0.0169
**Outpatient visits**				
Patients with an outpatient visit, (%)	5,328 (56.1%)	2,475 (59.2%)	2,853 (53.7%)	
Number of outpatient visits PPPY (95% CI)	3.83 (3.69-3.97)	3.79 (3.59-3.99)	3.87 (3.67-4.07)	0.5826
Outpatient costs $ PPPY (95% CI)	$15,397 ($14,450-$16,406)	$17,228 ($15,811-$18,772)	$13,869 ($12,627-$15,233)	0.0008
**Emergency room visits**				
Patients with ER visit, n (%)	948 (10.0%)	421 (10.1%)	527 (9.9%)	
Number of ED visits	0.20 (0.18-0.23)	0.21 (0.18-0.25)	0.20 (0.17-0.22)	0.3874
ED costs	$448 ($400-$502)	$467 ($390-$559)	$431 ($374-$498)	0.5049
**Urgent Care (n [PPPY], 95% CI)**†				
Patients with UC visits, n (%)	4,635 (48.8%)	2,123 (50.8%)	2,512 (47.3%)	
Number of UC visits	2.14 (2.07-2.22)	2.06 (1.96-2.17)	2.21 (2.12-2.31)	0.0380
UC costs	$599 ($577-$623)	$596 ($564-$630)	$602 ($571-$635)	0.7923
**Pharmacy (n [PPPY], 95% CI)**†				
Patients with at least one pharmacy record, n (%)	8,372 (88.2%)	3,618 (86.5%)	4,754 (89.5%)	
Number of distinct drugs	0.91 (0.89-0.92)	0.83 (0.8-0.85)	0.98 (0.95-1)	<0.0001
Pharmacy costs	$145 ($136-$155)	$134 ($119-$151)	$154 ($142-$166)	0.0572

Healthcare costs are presented as PPPY $USD 2022. Healthcare resource utilization is presented as PPPY.
CI, confidence interval; HCM, hypertrophic cardiomyopathy; ER, emergency room; PPPY, per person per year; UC, urgent care.

Supplementary Table 2. Unadjusted All-Cause Healthcare Resource Utilization and Costs

	Overall (n=9,490)	Male (n=4,181)	Female (n=5,309)	*p*-value
**Total cost, PPPY (95% CI)**	$51,835 ($48,811-$55,047)	$54,327 ($49,775-$59,294)	$49,847 ($45,892-$54,142)	0.1611
**Hospitalizations**				
Patients with a hospitalization, n (%)	2,862 (30.2%)	1,228 (29.4%)	1,634 (30.8%)	
Number of hospitalizations, PPPY (95% CI)	0.54 (0.52-0.56)	0.48 (0.45-0.52)	0.58 (0.55-0.62)	<0.0001
Hospitalization costs $ PPPY (95% CI)	$4,779 ($4,488-$5,090)	$4,719 ($4,294-$5,186)	$4,832 ($4,440-$5,259)	0.7122
Length of stay, per hospitalization Mean days (95% CI)	5.26 (5.03-5.5)	5.06 (4.7-5.44)	5.41 (5.11-5.73)	0.1589
**Outpatient visits**				
Patients with an outpatient visit, (%)	7,815 (82.3%)	3,461 (82.8%)	4,354 (82.0%)	
Number of outpatient visits PPPY (95% CI)	11.52 (11.13-11.91)	11.18 (10.65-11.74)	11.78 (11.25-12.34)	0.1271
Outpatient costs $ PPPY (95% CI)	$37,679 ($34,806-$40,789)	$40,346 ($36,031-$45,178)	$35,553 ($31,817-$39,729)	0.1179
**Emergency room visits**				
Patients with an ER visit, n (%)	2,665 (28.1%)	1,148 (27.5%)	1,517 (28.6%)	
Number of ED visits	0.76 (0.71-0.8)	0.77 (0.69-0.85)	0.75 (0.69-0.8)	0.6913
ED costs $ PPPY (95% CI)	$1,603 ($1,490-$1,725)	$1,636 ($1,454-$1,841)	$1,573 ($1,436-$1,723)	0.6038
**Urgent Care visits**				
Patients with an UC visit, n (%)	6,697 (70.6%)	2,965 (70.9%)	3,732 (70.3%)	
Number of UC visits PPPY (95% CI)	5.19 (5.07-5.32)	4.93 (4.75-5.12)	5.40 (5.23-5.57)	0.0003
UC costs $ PPPY (95% CI)	$1,321 ($1,282-$1,360)	$1,307 ($1,249-$1,369)	$1,331 ($1,282-$1,383)	0.5482
**Pharmacy**				
Patients with at least one pharmacy record, n (%)	9,026 (95.1%)	3,952 (94.5%)	5,074 (95.6%)	
Number of distinct drugs PPPY (95% CI)	7.87 (7.73-8.01)	6.86 (6.68-7.05)	8.76 (8.54-8.98)	<0.0001
Pharmacy costs $ PPPY (95% CI)	$3,092 ($2,904-$3,292)	$3,106 ($2,815-$3,426)	$3,081 ($2,842-$3,340)	0.9020

Healthcare costs are presented as PPPY $USD 2022. Healthcare resource utilization is presented as PPPY.
CI, confidence interval; HCM, hypertrophic cardiomyopathy; ER, emergency room; PPPY, per person per year; UC, urgent care.

Supplementary Table 3. All-cause cost models result

Setting	Parameter	Level	Estimate	SE	Lower CL	Upper CL	Z	ProbZ
All-cause total	Intercept		9.4933	0.3329	8.8408	10.1458	28.52	<.0001
	Sex	Male	0.0068	0.0632	-0.1171	0.1308	0.11	0.914
	Region	Midwest	-0.1513	0.1066	-0.3603	0.0576	-1.42	0.1558
	Region	Northeast	0.0187	0.1132	-0.2031	0.2405	0.17	0.8688
	Region	South	-0.0766	0.1059	-0.2841	0.1309	-0.72	0.4695
	Region	Unknown	0.1669	0.3642	-0.5469	0.8807	0.46	0.6467
	Insurance plan type	Non-commercial	-0.0752	0.0587	-0.1904	0.0399	-1.28	0.2002
	Congestive Heart Failure	No	-0.3147	0.0694	-0.4507	-0.1787	-4.53	<.0001
	Hypertension 2 diagnosis	No	-0.1703	0.0627	-0.2931	-0.0475	-2.72	0.0066
	Stress cardiomyopathy	No	0.6117	0.2166	0.1872	1.0362	2.82	0.0047
	Coronary artery disease	No	-0.2388	0.0632	-0.3626	-0.115	-3.78	0.0002
	Atrial Fibrillation	No	-0.1216	0.0769	-0.2724	0.0292	-1.58	0.114
	Atrial Flutter	No	-0.4092	0.1293	-0.6627	-0.1557	-3.16	0.0016
	Ventricular Fibrillation	No	0.2201	0.1312	-0.0371	0.4773	1.68	0.0935
	Ventricular Tachycardia	No	-0.2261	0.0777	-0.3785	-0.0737	-2.91	0.0036
	Chronic Pulmonary Disease	No	-0.2123	0.0662	-0.3421	-0.0826	-3.21	0.0013
	Obesity	No	-0.0813	0.0591	-0.1971	0.0346	-1.37	0.1692
	Valvular Disease	No	-0.0089	0.0613	-0.129	0.1112	-0.15	0.8844
	Age (index treatment)		-0.0093	0.0024	-0.0139	-0.0047	-3.94	<.0001
Hospitalization	Intercept		7.2308	0.497	6.2566	8.2049	14.55	<.0001
	Sex	Male	-0.0251	0.0764	-0.1748	0.1247	-0.33	0.7427
	Region	Midwest	0.513	0.1184	0.281	0.7451	4.33	<.0001
	Region	Northeast	0.2733	0.1214	0.0353	0.5113	2.25	0.0244
	Region	South	0.2723	0.1178	0.0414	0.5032	2.31	0.0208
	Region	Unknown	0.7108	0.4042	-0.0813	1.5029	1.76	0.0786
	Insurance plan type	Non-commercial	-0.3874	0.0747	-0.5337	-0.2411	-5.19	<.0001
	Congestive Heart Failure	No	-0.43	0.078	-0.5829	-0.2771	-5.51	<.0001
	Hypertension 2 diagnosis	No	-0.0115	0.08	-0.1683	0.1453	-0.14	0.8857
	Stress cardiomyopathy	No	0.8291	0.3856	0.0735	1.5848	2.15	0.0315
	Coronary artery disease	No	-0.5669	0.0831	-0.7298	-0.4039	-6.82	<.0001
	Atrial Fibrillation	No	-0.226	0.0868	-0.3961	-0.0559	-2.6	0.0092
	Atrial Flutter	No	-0.2906	0.1479	-0.5805	-0.0007	-1.96	0.0495
	Ventricular Fibrillation	No	0.0118	0.1945	-0.3695	0.3931	0.06	0.9516
	Ventricular Tachycardia	No	0.1173	0.0959	-0.0707	0.3053	1.22	0.2213
	Chronic Pulmonary Disease	No	-0.3149	0.0866	-0.4846	-0.1451	-3.64	0.0003

	Obesity	No	-0.2182	0.0865	-0.3877	-0.0488	-2.52	0.0116
	Valvular Disease	No	-0.9024	0.0751	-1.0496	-0.7551	-12.01	<.0001
	Age (index treatment)		-0.0092	0.0028	-0.0148	-0.0037	-3.26	0.0011
Outpatient	Intercept		9.1386	0.4297	8.2963	9.9809	21.27	<.0001
	Sex	Male	0.022	0.0823	-0.1393	0.1834	0.27	0.7889
	Region	Midwest	-0.2949	0.1388	-0.5671	-0.0228	-2.12	0.0336
	Region	Northeast	-0.0442	0.1459	-0.3302	0.2417	-0.3	0.7618
	Region	South	-0.105	0.136	-0.3716	0.1616	-0.77	0.44
	Region	Unknown	0.2146	0.4234	-0.6153	1.0444	0.51	0.6123
	Insurance plan type	Non-commercial	-0.0779	0.075	-0.2249	0.0691	-1.04	0.2989
	Congestive Heart Failure	No	-0.3456	0.0893	-0.5206	-0.1706	-3.87	0.0001
	Hypertension 2 diagnosis	No	-0.2183	0.0826	-0.3802	-0.0564	-2.64	0.0082
	Stress cardiomyopathy	No	0.7739	0.2809	0.2234	1.3244	2.76	0.0059
	Coronary artery disease	No	-0.1931	0.0819	-0.3535	-0.0326	-2.36	0.0183
	Atrial Fibrillation	No	-0.1257	0.0992	-0.3202	0.0687	-1.27	0.205
	Atrial Flutter	No	-0.5009	0.1628	-0.82	-0.1818	-3.08	0.0021
	Ventricular Fibrillation	No	0.2543	0.1627	-0.0646	0.5732	1.56	0.118
	Ventricular Tachycardia	No	-0.3402	0.0945	-0.5254	-0.155	-3.6	0.0003
	Chronic Pulmonary Disease	No	-0.1707	0.0883	-0.3438	0.0024	-1.93	0.0533
	Obesity	No	-0.0417	0.0794	-0.1973	0.1139	-0.53	0.5995
	Valvular Disease	No	0.0998	0.0803	-0.0577	0.2573	1.24	0.2141
	Age (index treatment)		-0.0098	0.0031	-0.0158	-0.0038	-3.21	0.0013
Emergency Room	Intercept		6.8843	0.5571	5.7925	7.9761	12.36	<.0001
	sex	Male	-0.0555	0.0715	-0.1956	0.0846	-0.78	0.4374
	Region	Midwest	-0.6036	0.1356	-0.8694	-0.3377	-4.45	<.0001
	Region	Northeast	-0.4682	0.1552	-0.7723	-0.164	-3.02	0.0026
	Region	South	-0.5379	0.132	-0.7967	-0.2791	-4.07	<.0001
	Region	Unknown	-0.7185	0.3336	-1.3722	-0.0647	-2.15	0.0313
	Insurance plan type	Non-commercial	0.4602	0.0834	0.2968	0.6236	5.52	<.0001
	Congestive Heart Failure	No	-0.3019	0.0716	-0.4421	-0.1616	-4.22	<.0001
	Hypertension 2 diagnosis	No	-0.3575	0.0731	-0.5006	-0.2143	-4.89	<.0001
	Stress cardiomyopathy	No	0.5179	0.4283	-0.3215	1.3573	1.21	0.2265
	Coronary artery disease	No	-0.4129	0.0698	-0.5497	-0.2761	-5.91	<.0001
	Atrial Fibrillation	No	-0.0869	0.0736	-0.2312	0.0573	-1.18	0.2376
	Atrial Flutter	No	-0.3378	0.1357	-0.6038	-0.0718	-2.49	0.0128
	Ventricular Fibrillation	No	0.4681	0.2067	0.063	0.8732	2.26	0.0235
	Ventricular Tachycardia	No	0.029	0.0942	-0.1557	0.2137	0.31	0.7581
	Chronic Pulmonary Disease	No	-0.5646	0.0738	-0.7092	-0.42	-7.65	<.0001

	Obesity	No	-0.138	0.0744	-0.2839	0.0079	-1.85	0.0637
	Valvular Disease	No	-0.0165	0.0774	-0.1683	0.1353	-0.21	0.8311
	Age (index treatment)		-0.0234	0.0031	-0.0294	-0.0174	-7.63	<.0001
Urgent Care	Intercept		6.0512	0.2496	5.562	6.5404	24.24	<.0001
	Sex	Male	-0.0004	0.0311	-0.0613	0.0605	-0.01	0.9895
	Region	Midwest	0.097	0.0498	-0.0005	0.1945	1.95	0.0513
	Region	Northeast	0.2463	0.0502	0.1478	0.3447	4.9	<.0001
	Region	South	-0.0951	0.0465	-0.1861	-0.004	-2.05	0.0407
	Region	Unknown	0.117	0.1768	-0.2294	0.4635	0.66	0.5079
	Insurance plan type	Non-commercial	0.0908	0.0323	0.0276	0.1541	2.82	0.0049
	Congestive Heart Failure	No	-0.0885	0.0324	-0.1521	-0.0249	-2.73	0.0064
	Hypertension 2 diagnosis	No	-0.341	0.0312	-0.4022	-0.2797	-10.91	<.0001
	Stress cardiomyopathy	No	-0.2586	0.1769	-0.6054	0.0881	-1.46	0.1438
	Coronary artery disease	No	-0.1024	0.0344	-0.1698	-0.035	-2.98	0.0029
	Atrial Fibrillation	No	0.0016	0.0353	-0.0676	0.0709	0.05	0.9628
	Atrial Flutter	No	-0.1143	0.0642	-0.2401	0.0116	-1.78	0.0751
	Ventricular Fibrillation	No	-0.0651	0.1235	-0.307	0.1769	-0.53	0.5983
	Ventricular Tachycardia	No	-0.0475	0.0402	-0.1262	0.0313	-1.18	0.2374
	Chronic Pulmonary Disease	No	-0.2279	0.0362	-0.2988	-0.1571	-6.3	<.0001
	Obesity	No	-0.1301	0.0338	-0.1964	-0.0638	-3.84	0.0001
	Valvular Disease	No	-0.1371	0.0302	-0.1962	-0.0779	-4.54	<.0001
	Age (index treatment)		-0.0062	0.0012	-0.0084	-0.0039	-5.32	<.0001
Pharmacy	Intercept		5.2841	0.4094	4.4816	6.0866	12.91	<.0001
	Sex	Male	0.0804	0.07	-0.0568	0.2175	1.15	0.2507
	Region	Midwest	-0.0225	0.1254	-0.2682	0.2233	-0.18	0.8578
	Region	Northeast	0.1766	0.1327	-0.0834	0.4366	1.33	0.1831
	Region	South	-0.0449	0.131	-0.3017	0.2119	-0.34	0.7318
	Region	Unknown	-0.2332	0.3606	-0.94	0.4736	-0.65	0.5178
	Insurance plan type	Non-commercial	0.4889	0.0831	0.326	0.6518	5.88	<.0001
	Congestive Heart Failure	No	-0.0699	0.072	-0.2111	0.0713	-0.97	0.3321
	Hypertension 2 diagnosis	No	-0.117	0.0665	-0.2474	0.0134	-1.76	0.0785
	Stress cardiomyopathy	No	-0.2494	0.3025	-0.8424	0.3435	-0.82	0.4097
	Coronary artery disease	No	-0.0614	0.0673	-0.1933	0.0705	-0.91	0.3614
	Atrial Fibrillation	No	-0.3407	0.0747	-0.487	-0.1944	-4.56	<.0001
	Atrial Flutter	No	0.0183	0.1019	-0.1815	0.2181	0.18	0.8577
	Ventricular Fibrillation	No	0.4102	0.1708	0.0755	0.7449	2.4	0.0163
	Ventricular Tachycardia	No	0.2936	0.0863	0.1244	0.4628	3.4	0.0007
	Chronic Pulmonary Disease	No	-0.2991	0.0666	-0.4296	-0.1686	-4.49	<.0001

	Obesity	No	-0.0769	0.0688	-0.2118	0.0581	-1.12	0.2642
	Valvular Disease	No	0.1878	0.078	0.0349	0.3407	2.41	0.0161
	Age (index treatment)		-0.0009	0.0023	-0.0054	0.0035	-0.42	0.6744

Supplementary Table 4. All-cause resource models result

Setting	Parameter	Level	Estimate	SE	Lower CL	Upper CL	Z	ProbZ
Number of hospitalizations	Intercept		-0.6811	0.3213	-1.3107	-0.0514	-2.12	0.034
	Sex	Male	-0.164	0.0443	-0.2508	-0.0772	-3.7	0.0002
	Region	Midwest	-0.0832	0.0724	-0.225	0.0587	-1.15	0.2504
	Region	Northeast	-0.0777	0.0774	-0.2295	0.074	-1	0.3153
	Region	South	-0.1002	0.0704	-0.2382	0.0378	-1.42	0.1546
	Region	Unknown	0.1132	0.2748	-0.4253	0.6518	0.41	0.6803
	Insurance plan type	Non-commercial	0.1526	0.0474	0.0596	0.2456	3.22	0.0013
	Congestive Heart Failure	No	-0.4001	0.0453	-0.489	-0.3113	-8.83	<.0001
	Hypertension 2 diagnosis	No	-0.1573	0.0464	-0.2483	-0.0663	-3.39	0.0007
	Stress cardiomyopathy	No	-0.3284	0.2546	-0.8274	0.1706	-1.29	0.1971
	Coronary artery disease	No	-0.3756	0.047	-0.4676	-0.2835	-8	<.0001
	Atrial Fibrillation	No	-0.2318	0.0505	-0.3309	-0.1327	-4.59	<.0001
	Atrial Flutter	No	-0.2869	0.08	-0.4437	-0.1301	-3.59	0.0003
	Ventricular Fibrillation	No	-0.1861	0.1297	-0.4403	0.068	-1.44	0.1512
	Ventricular Tachycardia	No	0.0429	0.0625	-0.0796	0.1654	0.69	0.4927
	Chronic Pulmonary Disease	No	-0.4045	0.0482	-0.4989	-0.3101	-8.4	<.0001
	Obesity	No	-0.071	0.0507	-0.1703	0.0284	-1.4	0.1614
	Valvular Disease	No	-0.3565	0.0459	-0.4466	-0.2665	-7.76	<.0001
	Age (index treatment)		-0.0083	0.0018	-0.0119	-0.0048	-4.61	<.0001
Length of stay, per hospitalization	Intercept		1.5499	0.2022	1.1535	1.9463	7.66	<.0001
	Sex	Male	-0.0824	0.0551	-0.1903	0.0255	-1.5	0.1345
	Region	Midwest	0.0908	0.0645	-0.0357	0.2172	1.41	0.1594
	Region	Northeast	0.1053	0.0643	-0.0207	0.2313	1.64	0.1013
	Region	South	0.1124	0.0646	-0.0143	0.2391	1.74	0.0821
	Region	Unknown	0.1645	0.2086	-0.2443	0.5733	0.79	0.4303
	Insurance plan type	Non-commercial	-0.0413	0.0453	-0.1301	0.0475	-0.91	0.362
	Congestive Heart Failure	No	-0.1922	0.0416	-0.2737	-0.1107	-4.62	<.0001
	Hypertension 2 diagnosis	No	0.0479	0.0435	-0.0374	0.1332	1.1	0.2708
	Stress cardiomyopathy	No	0.4971	0.0992	0.3028	0.6915	5.01	<.0001
	Coronary artery disease	No	0.0368	0.043	-0.0476	0.1211	0.85	0.393
	Atrial Fibrillation	No	-0.0607	0.0457	-0.1502	0.0288	-1.33	0.1837
	Atrial Flutter	No	-0.1087	0.0764	-0.2583	0.041	-1.42	0.1547
	Ventricular Fibrillation	No	-0.1527	0.138	-0.4232	0.1178	-1.11	0.2685
	Ventricular Tachycardia	No	-0.0527	0.0618	-0.1738	0.0684	-0.85	0.3937
	Chronic Pulmonary Disease	No	0.0605	0.0456	-0.0288	0.1498	1.33	0.1842

	Obesity	No	-0.028	0.0536	-0.133	0.077	-0.52	0.6009
	Valvular Disease	No	-0.1798	0.0469	-0.2717	-0.0879	-3.83	0.0001
	Age (index treatment)		-0.0002	0.0019	-0.0039	0.0036	-0.08	0.9368
Number of outpatient visits	Intercept		1.1275	0.2285	0.6797	1.5754	4.93	<.0001
	Sex	Male	-0.0565	0.0347	-0.1245	0.0116	-1.63	0.104
	Region	Midwest	0.0924	0.0624	-0.03	0.2148	1.48	0.139
	Region	Northeast	0.1175	0.066	-0.0119	0.2468	1.78	0.0751
	Region	South	-0.0697	0.0629	-0.1931	0.0536	-1.11	0.2679
	Region	Unknown	0.1695	0.2908	-0.4005	0.7394	0.58	0.56
	Insurance plan type	Non-commercial	0.0764	0.0375	0.0029	0.1499	2.04	0.0415
	Congestive Heart Failure	No	-0.2442	0.0379	-0.3186	-0.1699	-6.44	<.0001
	Hypertension 2 diagnosis	No	-0.325	0.0364	-0.3963	-0.2536	-8.92	<.0001
	Stress cardiomyopathy	No	-0.0202	0.1812	-0.3754	0.335	-0.11	0.9113
	Coronary artery disease	No	-0.163	0.0365	-0.2344	-0.0915	-4.47	<.0001
	Atrial Fibrillation	No	-0.107	0.0427	-0.1907	-0.0233	-2.51	0.0122
	Atrial Flutter	No	-0.1723	0.0773	-0.3238	-0.0209	-2.23	0.0257
	Ventricular Fibrillation	No	0.0301	0.088	-0.1424	0.2025	0.34	0.7324
	Ventricular Tachycardia	No	-0.0477	0.0449	-0.1356	0.0402	-1.06	0.2874
	Chronic Pulmonary Disease	No	-0.1577	0.0373	-0.2307	-0.0847	-4.23	<.0001
	Obesity	No	-0.1038	0.0372	-0.1766	-0.0309	-2.79	0.0052
	Valvular Disease	No	-0.1144	0.0348	-0.1826	-0.0462	-3.29	0.001
	Age (index treatment)		-0.005	0.0013	-0.0075	-0.0024	-3.84	0.0001
Number of ER visits	Intercept		-0.6446	0.4464	-1.5196	0.2304	-1.44	0.1488
	Sex	Male	-0.0421	0.0598	-0.1594	0.0752	-0.7	0.482
	Region	Midwest	-0.0872	0.0925	-0.2684	0.094	-0.94	0.3454
	Region	Northeast	-0.0251	0.1046	-0.2302	0.1799	-0.24	0.8102
	Region	South	-0.1342	0.0911	-0.3128	0.0444	-1.47	0.1408
	Region	Unknown	0.1059	0.2802	-0.4434	0.6551	0.38	0.7056
	Insurance plan type	Non-commercial	0.5086	0.0643	0.3825	0.6347	7.9	<.0001
	Congestive Heart Failure	No	-0.2619	0.0627	-0.3849	-0.139	-4.18	<.0001
	Hypertension 2 diagnosis	No	-0.4245	0.0639	-0.5497	-0.2992	-6.64	<.0001
	Stress cardiomyopathy	No	0.2499	0.3545	-0.4448	0.9447	0.71	0.4808
	Coronary artery disease	No	-0.3968	0.058	-0.5104	-0.2832	-6.85	<.0001
	Atrial Fibrillation	No	-0.1748	0.0586	-0.2897	-0.0599	-2.98	0.0029
	Atrial Flutter	No	-0.3332	0.1245	-0.5771	-0.0892	-2.68	0.0074
	Ventricular Fibrillation	No	0.3591	0.1875	-0.0084	0.7265	1.92	0.0555
	Ventricular Tachycardia	No	0.0245	0.082	-0.1363	0.1852	0.3	0.7653
	Chronic Pulmonary Disease	No	-0.5291	0.0662	-0.6589	-0.3993	-7.99	<.0001

	Obesity	No	-0.1085	0.067	-0.2399	0.0228	-1.62	0.1052
	Valvular Disease	No	0.0789	0.0605	-0.0396	0.1975	1.3	0.192
	Age (index treatment)		-0.0267	0.0024	-0.0314	-0.022	-11.2	<.0001
Number of UC visits	Intercept		0.2813	0.213	-0.1361	0.6987	1.32	0.1865
	Sex	Male	-0.0467	0.0253	-0.0963	0.0028	-1.85	0.0647
	Region	Midwest	0.1214	0.0417	0.0397	0.2031	2.91	0.0036
	Region	Northeast	0.1534	0.0433	0.0685	0.2383	3.54	0.0004
	Region	South	-0.0061	0.0403	-0.0851	0.073	-0.15	0.8806
	Region	Unknown	0.1626	0.1577	-0.1464	0.4716	1.03	0.3024
	Insurance plan type	Non-commercial	0.1249	0.0264	0.0731	0.1767	4.72	<.0001
	Congestive Heart Failure	No	-0.1031	0.026	-0.154	-0.0521	-3.97	<.0001
	Hypertension 2 diagnosis	No	-0.3976	0.0253	-0.4473	-0.348	-15.69	<.0001
	Stress cardiomyopathy	No	-0.3001	0.1702	-0.6336	0.0334	-1.76	0.0778
	Coronary artery disease	No	-0.0703	0.0265	-0.1222	-0.0184	-2.66	0.0079
	Atrial Fibrillation	No	0.0047	0.0284	-0.051	0.0604	0.17	0.8688
	Atrial Flutter	No	-0.0742	0.0535	-0.1791	0.0307	-1.39	0.1656
	Ventricular Fibrillation	No	-0.0134	0.0921	-0.1939	0.1671	-0.15	0.8841
	Ventricular Tachycardia	No	-0.0437	0.0343	-0.1108	0.0234	-1.28	0.2021
	Chronic Pulmonary Disease	No	-0.2079	0.0277	-0.2622	-0.1537	-7.52	<.0001
	Obesity	No	-0.1632	0.0282	-0.2185	-0.1079	-5.78	<.0001
	Valvular Disease	No	-0.0966	0.0242	-0.1441	-0.0491	-3.98	<.0001
	Age (index treatment)		-0.0035	0.0009	-0.0053	-0.0016	-3.73	0.0002
Number of distinct generic drugs	Intercept		-0.6359	0.1635	-0.9562	-0.3155	15.13	0.0001
	Sex	Male	-0.1501	0.0184	-0.1862	-0.114	66.54	<.0001
	Region	Midwest	-0.0266	0.0306	-0.0866	0.0334	0.75	0.3853
	Region	Northeast	-0.0212	0.031	-0.0818	0.0395	0.47	0.4944
	Region	South	0.0138	0.0293	-0.0437	0.0714	0.22	0.6371
	Region	Unknown	-0.3417	0.1477	-0.6312	-0.0523	5.35	0.0207
	Insurance plan type	Non-commercial	0.5396	0.0192	0.502	0.5772	790.61	<.0001
	Congestive Heart Failure	No	-0.0531	0.0193	-0.0909	-0.0154	7.6	0.0058
	Hypertension 2 diagnosis	No	-0.0334	0.0191	-0.0709	0.0041	3.05	0.0808
	Stress cardiomyopathy	No	-0.2207	0.1375	-0.4901	0.0488	2.58	0.1085
	Coronary artery disease	No	-0.0409	0.02	-0.0801	-0.0017	4.19	0.0407
	Atrial Fibrillation	No	-0.0167	0.0215	-0.0588	0.0254	0.6	0.4373
	Atrial Flutter	No	-0.0956	0.04	-0.1741	-0.0172	5.72	0.0168
	Ventricular Fibrillation	No	0.2429	0.0621	0.1212	0.3647	15.29	<.0001
	Ventricular Tachycardia	No	0.281	0.0251	0.2318	0.3302	125.31	<.0001
	Chronic Pulmonary Disease	No	-0.28	0.0215	-0.322	-0.2379	170.15	<.0001

	Obesity	No	-0.0945	0.0218	-0.1373	-0.0517	18.77	<.0001
	Valvular Disease	No	0.1522	0.0184	0.1161	0.1883	68.31	<.0001
	Age (index treatment)		0.0005	0.0007	-0.001	0.0019	0.41	0.5221

Supplementary Table 5. HCM related cost models result

Setting	Parameter	Level	Estimate	SE	Lower CL	Upper CL	Z	ProbZ
HCM related total	Intercept		8.5893	0.3883	7.8282	9.3504	22.12	<.0001
	Sex	Male	-0.0173	0.0572	-0.1294	0.0949	-0.3	0.7629
	Region	Midwest	0.0116	0.0902	-0.1652	0.1884	0.13	0.8977
	Region	Northeast	0.0655	0.0986	-0.1278	0.2588	0.66	0.5063
	Region	South	0.0547	0.0948	-0.1312	0.2406	0.58	0.5639
	Region	Unknown	-0.1187	0.2523	-0.6131	0.3758	-0.47	0.638
	Insurance plan type	Non-commercial	-0.3842	0.0566	-0.4952	-0.2731	-6.78	<.0001
	Congestive Heart Failure	No	-0.2037	0.0618	-0.3247	-0.0826	-3.3	0.001
	Hypertension 2 diagnosis	No	-0.0254	0.0576	-0.1383	0.0874	-0.44	0.6585
	Stress cardiomyopathy	No	1.2089	0.3133	0.5948	1.8229	3.86	0.0001
	Coronary artery disease	No	-0.2129	0.0632	-0.3369	-0.089	-3.37	0.0008
	Atrial Fibrillation	No	-0.1115	0.071	-0.2507	0.0277	-1.57	0.1165
	Atrial Flutter	No	-0.3308	0.1106	-0.5476	-0.114	-2.99	0.0028
	Ventricular Fibrillation	No	0.1486	0.1528	-0.1509	0.448	0.97	0.3308
	Ventricular Tachycardia	No	-0.5558	0.0755	-0.7039	-0.4078	-7.36	<.0001
	Chronic Pulmonary Disease	No	-0.1152	0.0688	-0.2501	0.0197	-1.67	0.0943
	Obesity	No	-0.1409	0.0626	-0.2636	-0.0182	-2.25	0.0245
	Valvular Disease	No	-0.1408	0.0591	-0.2565	-0.025	-2.38	0.0171
	Age (index treatment)		-0.0164	0.0021	-0.0204	-0.0123	-7.95	<.0001
Hospitalization	Intercept		5.5761	0.6468	4.3084	6.8438	8.62	<.0001
	Sex	Male	-0.0446	0.102	-0.2445	0.1553	-0.44	0.6617
	Region	Midwest	0.575	0.1701	0.2415	0.9084	3.38	0.0007
	Region	Northeast	0.0741	0.1804	-0.2796	0.4277	0.41	0.6814
	Region	South	0.2488	0.1726	-0.0894	0.5871	1.44	0.1493
	Region	Unknown	1.0258	0.5407	-0.0339	2.0855	1.9	0.0578
	Insurance plan type	Non-commercial	-0.3813	0.099	-0.5753	-0.1873	-3.85	0.0001
	Congestive Heart Failure	No	-0.2999	0.1077	-0.511	-0.0889	-2.79	0.0053
	Hypertension 2 diagnosis	No	0.066	0.1151	-0.1595	0.2916	0.57	0.5661
	Stress cardiomyopathy	No	1.5969	0.4503	0.7144	2.4794	3.55	0.0004
	Coronary artery disease	No	-0.6295	0.1213	-0.8671	-0.3918	-5.19	<.0001
	Atrial Fibrillation	No	-0.168	0.1117	-0.3869	0.0508	-1.5	0.1324
	Atrial Flutter	No	-0.0834	0.2334	-0.5407	0.374	-0.36	0.7209
	Ventricular Fibrillation	No	0.3817	0.2823	-0.1715	0.9349	1.35	0.1763
	Ventricular Tachycardia	No	0.2394	0.1307	-0.0168	0.4956	1.83	0.067
	Chronic Pulmonary Disease	No	-0.3195	0.1249	-0.5642	-0.0748	-2.56	0.0105

	Obesity	No	-0.1397	0.1149	-0.3649	0.0854	-1.22	0.2239
	Valvular Disease	No	-1.0156	0.1031	-1.2178	-0.8134	-9.85	<.0001
	Age (index treatment)		-0.0163	0.0036	-0.0234	-0.0092	-4.52	<.0001
Outpatient	Intercept		8.5309	0.4599	7.6295	9.4324	18.55	<.0001
	Sex	Male	0.0175	0.071	-0.1217	0.1567	0.25	0.8053
	Region	Midwest	-0.1196	0.1125	-0.3402	0.1009	-1.06	0.2877
	Region	Northeast	0.0457	0.1226	-0.1947	0.286	0.37	0.7097
	Region	South	0.0335	0.1185	-0.1988	0.2658	0.28	0.7775
	Region	Unknown	-0.2714	0.3135	-0.8858	0.343	-0.87	0.3866
	Insurance plan type	Non-commercial	-0.3995	0.069	-0.5348	-0.2642	-5.79	<.0001
	Congestive Heart Failure	No	-0.2058	0.0747	-0.3523	-0.0593	-2.75	0.0059
	Hypertension 2 diagnosis	No	-0.0624	0.0704	-0.2005	0.0756	-0.89	0.3756
	Stress cardiomyopathy	No	1.1591	0.3701	0.4338	1.8844	3.13	0.0017
	Coronary artery disease	No	-0.1369	0.0778	-0.2894	0.0156	-1.76	0.0784
	Atrial Fibrillation	No	-0.1292	0.0877	-0.301	0.0427	-1.47	0.1407
	Atrial Flutter	No	-0.419	0.1336	-0.6808	-0.1571	-3.14	0.0017
	Ventricular Fibrillation	No	0.1133	0.1805	-0.2405	0.4671	0.63	0.5303
	Ventricular Tachycardia	No	-0.7059	0.0862	-0.8749	-0.5369	-8.19	<.0001
	Chronic Pulmonary Disease	No	-0.092	0.0862	-0.2609	0.077	-1.07	0.2859
	Obesity	No	-0.1331	0.0794	-0.2887	0.0225	-1.68	0.0935
	Valvular Disease	No	0.0342	0.0733	-0.1096	0.1779	0.47	0.6415
	Age (index treatment)		-0.0167	0.0025	-0.0216	-0.0118	-6.67	<.0001
Emergency Room	Intercept		4.2372	1.0428	2.1933	6.2811	4.06	<.0001
	Sex	Male	-0.1581	0.1141	-0.3817	0.0656	-1.39	0.166
	Region	Midwest	-0.3503	0.1732	-0.6897	-0.0109	-2.02	0.0431
	Region	Northeast	-0.1871	0.1946	-0.5686	0.1943	-0.96	0.3363
	Region	South	-0.3851	0.1631	-0.7049	-0.0654	-2.36	0.0182
	Region	Unknown	-0.2222	0.4429	-1.0903	0.6459	-0.5	0.6159
	Insurance plan type	Non-commercial	0.4469	0.1247	0.2025	0.6912	3.58	0.0003
	Congestive Heart Failure	No	-0.4562	0.1145	-0.6805	-0.2318	-3.99	<.0001
	Hypertension 2 diagnosis	No	-0.0912	0.117	-0.3206	0.1381	-0.78	0.4357
	Stress cardiomyopathy	No	2.2535	0.8757	0.537	3.9699	2.57	0.0101
	Coronary artery disease	No	-0.4705	0.1221	-0.7098	-0.2312	-3.85	0.0001
	Atrial Fibrillation	No	-0.0481	0.1274	-0.2978	0.2017	-0.38	0.7061
	Atrial Flutter	No	-0.4223	0.2021	-0.8184	-0.0262	-2.09	0.0367
	Ventricular Fibrillation	No	0.4316	0.2804	-0.1181	0.9812	1.54	0.1238
	Ventricular Tachycardia	No	0.066	0.144	-0.2161	0.3482	0.46	0.6465
	Chronic Pulmonary Disease	No	-0.3806	0.1289	-0.6332	-0.1281	-2.95	0.0031

	Obesity	No	-0.1413	0.1206	-0.3776	0.0951	-1.17	0.2414
	Valvular Disease	No	-0.1467	0.1134	-0.3689	0.0755	-1.29	0.1956
	Age (index treatment)		-0.0325	0.0043	-0.0409	-0.0242	-7.61	<.0001
Urgent Care	Intercept		5.5173	0.2934	4.9422	6.0924	18.8	<.0001
	Sex	Male	-0.0423	0.0411	-0.1229	0.0384	-1.03	0.304
	Region	Midwest	0.0936	0.0638	-0.0315	0.2187	1.47	0.1425
	Region	Northeast	0.2157	0.0689	0.0806	0.3507	3.13	0.0017
	Region	South	-0.072	0.0632	-0.1959	0.0518	-1.14	0.2543
	Region	Unknown	0.3058	0.2727	-0.2286	0.8402	1.12	0.2621
	Insurance plan type	Non-commercial	0.1055	0.0442	0.019	0.1921	2.39	0.0169
	Congestive Heart Failure	No	0.0099	0.0464	-0.0811	0.1008	0.21	0.8318
	Hypertension 2 diagnosis	No	-0.2547	0.0422	-0.3374	-0.1721	-6.04	<.0001
	Stress cardiomyopathy	No	-0.3563	0.2121	-0.7719	0.0594	-1.68	0.093
	Coronary artery disease	No	0.0121	0.0472	-0.0803	0.1046	0.26	0.7969
	Atrial Fibrillation	No	0.0821	0.0498	-0.0155	0.1797	1.65	0.0992
	Atrial Flutter	No	0.0642	0.0856	-0.1036	0.232	0.75	0.4537
	Ventricular Fibrillation	No	-0.1457	0.1391	-0.4184	0.127	-1.05	0.2949
	Ventricular Tachycardia	No	-0.1585	0.0567	-0.2697	-0.0473	-2.79	0.0052
	Chronic Pulmonary Disease	No	-0.0945	0.0476	-0.1877	-0.0013	-1.99	0.0469
	Obesity	No	-0.1061	0.0458	-0.1958	-0.0164	-2.32	0.0204
	Valvular Disease	No	-0.2061	0.0411	-0.2866	-0.1255	-5.01	<.0001
	Age (index treatment)		-0.0135	0.0015	-0.0163	-0.0106	-9.25	<.0001
Pharmacy	Intercept		0.9835	0.4038	0.1921	1.775	2.44	0.0149
	Sex	Male	-0.1373	0.069	-0.2725	-0.0021	-1.99	0.0465
	Region	Midwest	0.2261	0.0999	0.0303	0.4218	2.26	0.0236
	Region	Northeast	0.4545	0.1133	0.2323	0.6766	4.01	<.0001
	Region	South	0.0527	0.0975	-0.1384	0.2437	0.54	0.5889
	Region	Unknown	-0.1695	0.2668	-0.6925	0.3535	-0.64	0.5253
	Insurance plan type	Non-commercial	0.6366	0.0768	0.486	0.7872	8.28	<.0001
	Congestive Heart Failure	No	0.135	0.0771	-0.016	0.286	1.75	0.0798
	Hypertension 2 diagnosis	No	-0.1423	0.0658	-0.2712	-0.0133	-2.16	0.0306
	Stress cardiomyopathy	No	0.6256	0.1992	0.2351	1.0161	3.14	0.0017
	Coronary artery disease	No	0.1729	0.0756	0.0247	0.3211	2.29	0.0222
	Atrial Fibrillation	No	-0.0074	0.0758	-0.156	0.1412	-0.1	0.9226
	Atrial Flutter	No	0.0268	0.1292	-0.2265	0.2801	0.21	0.8358
	Ventricular Fibrillation	No	0.0433	0.3171	-0.5783	0.6649	0.14	0.8913
	Ventricular Tachycardia	No	0.4213	0.0882	0.2484	0.5942	4.78	<.0001
	Chronic Pulmonary Disease	No	-0.0234	0.0818	-0.1837	0.1368	-0.29	0.7745

	Obesity	No	-0.123	0.0848	-0.2891	0.0431	-1.45	0.1467
	Valvular Disease	No	0.1508	0.0686	0.0163	0.2853	2.2	0.028
	Age (index treatment)		-0.0048	0.0021	-0.0088	-0.0007	-2.3	0.0215

Supplementary Table 6. HCM related resource use models result

Setting	Parameter	Level	Estimate	SE	Lower CL	Upper CL	Z	ProbZ
Number of hospitalizations	Intercept		-2.2043	0.5387	-3.2602	-1.1484	-4.09	<.0001
	Sex	Male	-0.1932	0.0606	-0.3121	-0.0744	-3.19	0.0014
	Region	Midwest	0.0949	0.0953	-0.092	0.2817	0.99	0.3198
	Region	Northeast	-0.0395	0.1065	-0.2482	0.1693	-0.37	0.711
	Region	South	0.052	0.0937	-0.1316	0.2356	0.56	0.5788
	Region	Unknown	0.2387	0.316	-0.3807	0.8581	0.76	0.45
	Insurance plan type	Non-commercial	-0.0774	0.0636	-0.202	0.0471	-1.22	0.2231
	Congestive Heart Failure	No	-0.2941	0.0644	-0.4204	-0.1678	-4.56	<.0001
	Hypertension 2 diagnosis	No	-0.0378	0.063	-0.1614	0.0857	-0.6	0.5482
	Stress cardiomyopathy	No	0.3546	0.4599	-0.5468	1.2561	0.77	0.4407
	Coronary artery disease	No	-0.3803	0.0666	-0.5109	-0.2496	-5.71	<.0001
	Atrial Fibrillation	No	-0.2547	0.0718	-0.3955	-0.1139	-3.55	0.0004
	Atrial Flutter	No	-0.1105	0.1179	-0.3416	0.1207	-0.94	0.349
	Ventricular Fibrillation	No	-0.1254	0.1846	-0.4872	0.2364	-0.68	0.4969
	Ventricular Tachycardia	No	0.0183	0.0809	-0.1402	0.1768	0.23	0.8208
	Chronic Pulmonary Disease	No	-0.3187	0.0696	-0.4552	-0.1822	-4.58	<.0001
	Obesity	No	-0.0798	0.0693	-0.2156	0.056	-1.15	0.2493
	Valvular Disease	No	-0.5463	0.0624	-0.6687	-0.4239	-8.75	<.0001
	Age (index treatment)		-0.0136	0.0023	-0.0182	-0.009	-5.84	<.0001
Length of stay, per hospitalization	Intercept		1.4973	0.2972	0.9148	2.0797	5.04	<.0001
	Sex	Male	-0.1652	0.0729	-0.3081	-0.0223	-2.27	0.0235
	Region	Midwest	0.2327	0.0995	0.0376	0.4278	2.34	0.0194
	Region	Northeast	0.1425	0.0881	-0.0301	0.3152	1.62	0.1057
	Region	South	0.1462	0.0907	-0.0316	0.3241	1.61	0.1071
	Region	Unknown	0.1985	0.3056	-0.4004	0.7975	0.65	0.5158
	Insurance plan type	Non-commercial	-0.0021	0.0677	-0.1349	0.1306	-0.03	0.9748
	Congestive Heart Failure	No	-0.1406	0.058	-0.2543	-0.0269	-2.42	0.0154
	Hypertension 2 diagnosis	No	-0.0272	0.0659	-0.1563	0.1019	-0.41	0.6796
	Stress cardiomyopathy	No	0.4067	0.144	0.1244	0.689	2.82	0.0047
	Coronary artery disease	No	0.0894	0.0641	-0.0361	0.215	1.4	0.1627
	Atrial Fibrillation	No	-0.0973	0.0601	-0.215	0.0204	-1.62	0.1053
	Atrial Flutter	No	-0.303	0.1453	-0.5877	-0.0182	-2.09	0.037
	Ventricular Fibrillation	No	-0.1131	0.2459	-0.595	0.3688	-0.46	0.6456
	Ventricular Tachycardia	No	-0.0385	0.1023	-0.239	0.1619	-0.38	0.7064
	Chronic Pulmonary Disease	No	0.1185	0.0601	0.0007	0.2363	1.97	0.0487

	Obesity	No	0.0251	0.0735	-0.119	0.1691	0.34	0.7333
	Valvular Disease	No	-0.0946	0.0689	-0.2297	0.0406	-1.37	0.1701
	Age (index treatment)		0.0012	0.0032	-0.0051	0.0075	0.37	0.7143
Number of outpatient visits	Intercept		0.4584	0.3058	-0.1411	1.0578	1.5	0.1339
	Sex	Male	-0.0804	0.0389	-0.1566	-0.0042	-2.07	0.0387
	Region	Midwest	0.1719	0.0673	0.04	0.3038	2.55	0.0106
	Region	Northeast	0.1987	0.0703	0.061	0.3364	2.83	0.0047
	Region	South	-0.0146	0.0663	-0.1447	0.1154	-0.22	0.8254
	Region	Unknown	0.0306	0.2184	-0.3974	0.4586	0.14	0.8885
	Insurance plan type	Non-commercial	0.0397	0.0422	-0.043	0.1224	0.94	0.3471
	Congestive Heart Failure	No	-0.1486	0.0425	-0.2319	-0.0654	-3.5	0.0005
	Hypertension 2 diagnosis	No	-0.198	0.0398	-0.2761	-0.1199	-4.97	<.0001
	Stress cardiomyopathy	No	-0.0294	0.2463	-0.5122	0.4533	-0.12	0.9049
	Coronary artery disease	No	-0.0944	0.0432	-0.179	-0.0097	-2.19	0.0289
	Atrial Fibrillation	No	-0.0337	0.0463	-0.1243	0.057	-0.73	0.4669
	Atrial Flutter	No	-0.112	0.0824	-0.2735	0.0494	-1.36	0.1739
	Ventricular Fibrillation	No	-0.0437	0.1231	-0.2849	0.1975	-0.36	0.7225
	Ventricular Tachycardia	No	-0.2192	0.0486	-0.3144	-0.124	-4.51	<.0001
	Chronic Pulmonary Disease	No	-0.1138	0.0451	-0.2022	-0.0255	-2.53	0.0115
	Obesity	No	-0.1242	0.044	-0.2105	-0.0379	-2.82	0.0048
	Valvular Disease	No	-0.1805	0.0392	-0.2574	-0.1037	-4.61	<.0001
	Age (index treatment)		-0.0128	0.0014	-0.0155	-0.0101	-9.26	<.0001
Number of ER visits	Intercept		-2.3185	0.8109	-3.9077	-0.7293	-2.86	0.0042
	Sex	Male	-0.1097	0.0948	-0.2955	0.076	-1.16	0.247
	Region	Midwest	-0.0513	0.1385	-0.3227	0.2202	-0.37	0.7113
	Region	Northeast	0.1272	0.1569	-0.1803	0.4346	0.81	0.4175
	Region	South	-0.0738	0.1334	-0.3353	0.1877	-0.55	0.5803
	Region	Unknown	0.5138	0.4057	-0.2813	1.3089	1.27	0.2053
	Insurance plan type	Non-commercial	0.5076	0.1103	0.2914	0.7237	4.6	<.0001
	Congestive Heart Failure	No	-0.442	0.1067	-0.651	-0.2329	-4.14	<.0001
	Hypertension 2 diagnosis	No	-0.1966	0.1005	-0.3936	0.0004	-1.96	0.0505
	Stress cardiomyopathy	No	1.3443	0.7252	-0.0771	2.7656	1.85	0.0638
	Coronary artery disease	No	-0.4623	0.1012	-0.6607	-0.264	-4.57	<.0001
	Atrial Fibrillation	No	-0.0992	0.1071	-0.3091	0.1108	-0.93	0.3546
	Atrial Flutter	No	-0.3324	0.1802	-0.6857	0.0209	-1.84	0.0652
	Ventricular Fibrillation	No	0.014	0.2392	-0.4547	0.4828	0.06	0.9532
	Ventricular Tachycardia	No	-0.0096	0.1214	-0.2475	0.2284	-0.08	0.9371
	Chronic Pulmonary Disease	No	-0.3508	0.1023	-0.5513	-0.1503	-3.43	0.0006

	Obesity	No	-0.1384	0.1049	-0.3439	0.0672	-1.32	0.187
	Valvular Disease	No	-0.0697	0.0986	-0.2629	0.1236	-0.71	0.4799
	Age (index treatment)		-0.035	0.0035	-0.0418	-0.0282	-10.1	<.0001
Number of UC visits	Intercept		-0.3334	0.2785	-0.8793	0.2126	-1.2	0.2314
	Sex	Male	-0.0636	0.0355	-0.1332	0.0059	-1.79	0.0729
	Region	Midwest	0.1265	0.0572	0.0144	0.2387	2.21	0.027
	Region	Northeast	0.0695	0.0604	-0.0488	0.1878	1.15	0.2498
	Region	South	0.0181	0.0559	-0.0914	0.1275	0.32	0.7464
	Region	Unknown	0.2837	0.2121	-0.132	0.6994	1.34	0.1811
	Insurance plan type	Non-commercial	0.1425	0.0382	0.0677	0.2173	3.74	0.0002
	Congestive Heart Failure	No	-0.0375	0.038	-0.112	0.037	-0.99	0.3237
	Hypertension 2 diagnosis	No	-0.3137	0.0362	-0.3847	-0.2427	-8.66	<.0001
	Stress cardiomyopathy	No	-0.4198	0.2178	-0.8466	0.007	-1.93	0.0539
	Coronary artery disease	No	0.0487	0.0395	-0.0287	0.1262	1.23	0.2176
	Atrial Fibrillation	No	0.1152	0.042	0.033	0.1975	2.75	0.006
	Atrial Flutter	No	0.0266	0.0813	-0.1327	0.186	0.33	0.7434
	Ventricular Fibrillation	No	-0.066	0.1232	-0.3074	0.1754	-0.54	0.5919
	Ventricular Tachycardia	No	-0.1417	0.0469	-0.2337	-0.0498	-3.02	0.0025
	Chronic Pulmonary Disease	No	-0.0882	0.0416	-0.1696	-0.0067	-2.12	0.0339
	Obesity	No	-0.1183	0.0399	-0.1965	-0.0401	-2.96	0.003
	Valvular Disease	No	-0.1745	0.0347	-0.2424	-0.1065	-5.03	<.0001
	Age (index treatment)		-0.0101	0.0012	-0.0125	-0.0077	-8.18	<.0001
Number of distinct generic drugs	Intercept		-3.4505	0.1835	-3.8102	-3.0908	353.52	<.0001
	Sex	Male	-0.0693	0.021	-0.1105	-0.0281	10.88	0.001
	Region	Midwest	-0.1347	0.0338	-0.201	-0.0685	15.91	<.0001
	Region	Northeast	-0.1213	0.0339	-0.1878	-0.0548	12.78	0.0003
	Region	South	-0.0758	0.0321	-0.1387	-0.0129	5.58	0.0182
	Region	Unknown	-0.3974	0.1918	-0.7733	-0.0214	4.29	0.0383
	Insurance plan type	Non-commercial	0.7425	0.0251	0.6933	0.7917	874.66	<.0001
	Congestive Heart Failure	No	0.0245	0.022	-0.0185	0.0676	1.25	0.2638
	Hypertension 2 diagnosis	No	0.0179	0.0218	-0.0249	0.0608	0.67	0.4115
	Stress cardiomyopathy	No	-0.1803	0.138	-0.4508	0.0902	1.71	0.1914
	Coronary artery disease	No	0.007	0.0233	-0.0387	0.0527	0.09	0.765
	Atrial Fibrillation	No	0.0299	0.0252	-0.0194	0.0792	1.42	0.234
	Atrial Flutter	No	-0.0273	0.0492	-0.1237	0.0691	0.31	0.5783
	Ventricular Fibrillation	No	0.4373	0.0971	0.2469	0.6276	20.27	<.0001
	Ventricular Tachycardia	No	0.4586	0.0341	0.3918	0.5255	180.76	<.0001
	Chronic Pulmonary Disease	No	-0.14	0.0238	-0.1868	-0.0933	34.47	<.0001

	Obesity	No	-0.0322	0.0249	-0.0809	0.0165	1.68	0.1955
	Valvular Disease	No	0.1901	0.0214	0.1481	0.232	79.01	<.0001
	Age (index treatment)		-0.0029	0.0008	-0.0045	-0.0014	13.6	0.0002
